# Hepatic Stellate Cell-Derived Microvesicles Prevent Hepatocytes from Injury Induced by APAP/H_2_O_2_

**DOI:** 10.1155/2016/8357567

**Published:** 2016-04-28

**Authors:** Renwei Huang, Qunwen Pan, Xiaotang Ma, Yan Wang, Yaolong Liang, Bingyan Dai, Xiaorong Liao, Mingyi Li, Huilai Miao

**Affiliations:** ^1^Department of Hepatobiliary Surgery, Institute of Neurology, Affiliated Hospital of Guangdong Medical University, Zhanjiang 524001, China; ^2^Guangdong Key Laboratory of Age-Related Cardiac and Cerebral Diseases, Institute of Neurology, Affiliated Hospital of Guangdong Medical University, Zhanjiang 524001, China

## Abstract

Hepatic stellate cells (HSCs), previously described for liver-specific mesenchymal stem cells (MSCs), appear to contribute to liver regeneration. Microvesicles (MVs) are nanoscale membrane fragments, which can regulate target cell function by transferring contents from their parent cells. The aim of this study was to investigate the effect of HSC-derived MVs on xenobiotic-induced liver injury. Rat and human hepatocytes, BRL-3A and HL-7702, were used to build hepatocytes injury models by n-acetyl-p-aminophenol n-(APAP) or H_2_O_2_ treatment. MVs were prepared from human and rat HSCs, LX-2, and HST-T6 and, respectively, added to injured BRL-3A and HL-7702 hepatocytes. MTT assay was utilized to determine cell proliferation. Cell apoptosis was analyzed by flow cytometry and hoechst33258 staining. Western blot was used for analyzing the expression of activated caspase-3. Liver injury indicators, alanine aminotransferase (ALT), aspartate aminotransferase (AST), and lactate dehydrogenase (LDH) in culture medium were also assessed. Results showed that (1) HSC-MVs derived from LX-2 and HST-T6 were positive to CD90 and annexin V surface markers; (2) HSC-MVs dose-dependently improved the viability of hepatocytes in both injury models; (3) HSC-MVs dose-dependently inhibited the APAP/H_2_O_2_ induced hepatocytes apoptosis and activated caspase-3 expression and leakage of LDH, ALT, and AST. Our results demonstrate that HSC-derived MVs protect hepatocytes from toxicant-induced injury.

## 1. Introduction

The liver is an important organ of the body for metabolism of major nutrients and the major place for biotransformation. Multiple factors can lead to liver injury, such as genetic and metabolic factors, drugs, and virus [1, 2]. Chronic liver injury can ultimately lead to liver fibrosis and even hepatocellular carcinoma [3–5]. Although liver has the capacity to regenerate through the replication of mature functioning liver cells [6], acute and severe liver injury sometimes threatens life when cell death exceeds the self-regenerative capacity of the liver, such as liver failure [7]. Currently conventional drug treatment has little effect for mild liver damage. When severe liver injury such as liver failure happens, the most effective treatment is orthotopic liver transplantation. But this treatment is much limited by the shortage and high cost of donor organ and the need of immunosuppressive medication [8, 9]. Therefore, other options/approaches that can effectively improve liver regeneration and prevent liver injury are badly in need.

Hepatic stellate cells (HSCs) are liver-specific mesenchymal stem cells, which are located between hepatocytes and sinusoidal endothelial in the space of Disse [10]. Recent evidence suggests that HSCs play pivotal roles in liver physiology and fibrogenesis [11]. In homeostatic conditions, HSCs store vitamin A and maintain low proliferating activity [12]. When activated, HSCs could differentiate into hepatocyte-like cells and contribute to hepatocyte proliferation and liver regeneration [10, 13]. Moreover, activated hepatic stellate cells accelerated liver regeneration by producing angiogenic factors as well as cytokines, such as HGF, which can enhance the proliferation of liver progenitor cells and hepatocytes [14–16].

Cellular microvesicles (MVs), 0.1–1 *μ*m in size, are secreted by various types of cells which undergo stress, activation, or apoptosis. They can fuse with target cells and affect cell functions by transferring or delivering proteins and gene messages to the recipient cells. Recent studies have demonstrated that MVs derived from stem/progenitor/mesenchymal stromal cells (MSCs) possess therapeutic potentials similar to their parent cells [17, 18]. And MVs therapies may be more advantageous than MSCs for their less likely influenced systemic environment and smaller size, which makes it more easy to pass through tissue barriers [19, 20]. However, whether MVs derived from the HSCs could prevent liver injury as their parent cells remains unknown.

In this work, we will explore the effects of MVs derived from HSCs on the proliferation and apoptosis of hepatocytes through in vitro drug-induced injury models.

## 2. Materials and Methods

### 2.1. Cell Lines and Culture Conditions

LX-2 (Human Hepatic Stellate Cells) and HST-T6 (Mouse Hepatic Stellate Cells) were used to generate microvesicles. Meanwhile, the normal human hepatic cell (HL-7702) and mouse hepatic cell (BRL-3A) were set to establish liver injury models. The evidence in various liver injury models will help to strengthen the therapeutic effect of MVs.

All cells were obtained from Guangdong Joycer Biotech Co. Ltd. The cells were cultured on 100 mm cell culture dishes in DMEM (Hyclone), supplemented with 10% fetal bovine serum (FBS, GIBCO), 100 U/mL penicillin, and 100 U/mL streptomycin in a 37°C incubator with 5% CO2/95% air.

### 2.2. Injury Models of Hepatocytes

After the monolayer of cells became confluent in 96-well plate, BRL-3A and HL-7702 cells were treated with a range of concentration of APAP (3.2 mM, 4.8 mM, 6.4 mM, 8 mM, and 9.6 mM) or H_2_O_2_ (240 *μ*M, 360 *μ*M, 480 *μ*M, 600 *μ*M, and 720 *μ*M) in DMEM medium with 10% serum for 24 h [6]. After the 24 h treatment, the MTT assay in control, APAP, or H_2_O_2_ exposed cells was conducted. The EC50 values of APAP and H_2_O_2 _were determined and used for establishing in vitro hepatocyte injury models.

### 2.3. Preparation and Characterization of HSC-Derived MVs

MVs were generated from human stellate cells LX-2 (^Lx^HSC-MVs) and rat stellate cells HST-T6 (^T6^HSC-MVs) as previously described [21]. In brief, LX-2 and HST-T6 cells were cultured in 100 mm cell culture dishes. When cells grow to 80% confluence, LX-2 and HST-T6 cultures were washed with PBS and cultured in fresh growth culture medium for 24 h. Then the cell medium was collected and centrifuged at 300 g, 15 min, and followed by 2000 g, 30 min, to remove cells and cell debris. The cell-free culture medium was centrifuged at 20,000 g, 2 h to pellet MVs. The pelleted MVs were resuspended with 20 nm-filtered (Whatman, Pittsburgh, PA) phosphate-buffered saline (PBS).

HSCs have been shown to express classical mesenchymal markers (CD90) and can be quantified by flow cytometry [22]. For defining the HSC-MVs, samples were stained with PE-conjugated anti-mouse CD90, Alexa-488-labeled annexin V, 5 *μ*L, respectively. All antibodies were purchased from eBioscience (San Diego, CA). After incubation, labeled HSC-MVs were resuspended with PBS and analyzed under flow cytometry. The size and amount of the HSC-EMVs were measured by nanoparticle tracking analysis (Malvern, Britain).

### 2.4. In Vitro Cell Viability Assay

Cell viabilities of HL-7702 and BRL-3A were tested by MTT (3-[4,5-dimethylthiazyol-2yl]-2,5-diphenyltetrazolium bromide) (Sigma, 5 mg/mL) assay [23]. Cells were seeded at 2 × 10^3^/well in 96-well plate and cultured in 100 *μ*L DMEM medium (supplemented with 10% FBS). To establish in vitro hepatic injury model, HL-7702 cells were treated with 8 mM APAP or 600 *μ*M H_2_O_2_ and BRL-3A cells were treated with 6.4 mM APAP or 480 *μ*M H_2_O_2_. To determine the protective effect of HSC-MVs on injured hepatocytes proliferation, HL-7702 cells were cocultured with different concentration of ^Lx^HSC-MVs (2 × 10^6^/mL, 2 × 10^7^/mL, 2 × 10^8^/mL, resp., defined as L-^Lx^HSC-MVs, M-^Lx^HSC-MVs, and H-^Lx^MSC-MVs) when APAP/H_2_O_2_ was administered, and BRL-3A cells were cocultured with different concentration of ^T6^HSC-MVs (2 × 10^6^/mL, 2 × 10^7^/mL, 2 × 10^8^/mL, resp., defined as L-^T6^HSC-MVs, M-^T6^HSC-MVs, and H-^T6^HSC-MVs) when APAP/H_2_O_2_ was added. After incubation, cell viability test was carried out at 48 h time point.

PBS treated cells were used as the vehicle control groups. Positive groups were set for hepatocytes treated with APAP or H_2_O_2_ and without MVs incubation. After incubation of toxicants and different concentrations of MVs, the cell viability was evaluated with MTT assay. MTT solution (20 *μ*L) was added and incubated with cells for 4 h at 37°C; then 150 *μ*L DMSO was added to each well and incubated with the cells for 20 min at 37°C. The optical density (OD) value of cells was read at 490 nm in a microplate reader (BioTek). Measurement was carried out at 48 h. Cells in triplicate wells were examined at each time point, and the experiment was repeated three times. Results were calculated from the values obtained in three independent experiments.

### 2.5. Hoechst 33258 Staining Analysis of Cell Apoptosis

Cell apoptosis was analyzed by Hoechst 33258 staining as we previously described [23]. In brief, with 6-well plates, HL-7702 or BRL-3A cells were seeded at a density of 2 × 10^5^/well in 2 mL DMEM medium. Then the cells were treated with APAP/H_2_O_2_ and HSC-MVs. After 24 h treatment, cells were fixed, washed with PBS, and stained with hoechst33258 staining solution according to the manufacturer's instructions (Beyotime) and observed under a fluorescence microscope. Five independent fields were assessed for each well, and the average number of positive cells and total cells per field (magnification, 200x) were determined. The apoptosis rate of cells was defined as the ratio of positive cells versus total cells.

### 2.6. Flow Cytometry Analysis of Cell Apoptosis

HL-7702 and BRL-3A cells were treated as mentioned above. The apoptosis assay was conducted using an annexin V-PE/7-AAD apoptosis detection kit (BD Biosciences) as previously described [23]. Briefly, cells were washed with PBS, resuspended with 100 *μ*L 1 × annexin-binding buffer, incubated with 5 *μ*L PE-conjugated annexin V and 5 *μ*L 7-amino-actinomycin (7-AAD) for 15 min in the dark, and then analyzed by flow cytometry. Cells stained with both annexin V-PE and 7-AAD were considered to be late apoptotic HL-7702 or BRL-3A and cells stained only with annexin V-PE were considered to be early apoptotic cells [24]. The experiment was repeated for three times. And three plates per experiment were analyzed in each group.

### 2.7. Metabolites Detection of Hepatocytes

Alanine aminotransferase (ALT), aspartate aminotransferase (AST), and lactate dehydrogenase (LDH) are routinely measured for the detection of liver disruption [25, 26]. In the present study, LDH leakage was assessed by measuring the activity of LDH in the cell cultured media by LDH assay kit (Beyotime, China). Besides, the AST and ALT in the cell cultured media were also measured by the respective detection assay kits (Nanjing Jiancheng Bioengineering Institute, China). Briefly, HL-7702 and BRL-3A cells were placed in 6-well plates. After growing to 80% confluence, cells were treated as we described above. After 24 h treatment, culture medium of each group was collected for measuring the levels of LDH, AST, and ALT according to the manufactory instructions.

### 2.8. Western Blot Analysis

Proteins from HL-7702 cells and BRL-3A cells were extracted with lysis buffer. Protein lysates were electrophoresed through SDS-PAGE gel and transferred onto PVDF membranes. The membranes were blocked for 1 h and incubated with primary antibodies against caspase-3 (CST, USA) and *β*-actin (CST, USA) at 4°C overnight. After washing 3 times for 30 min with TBST, the immunoreactivity was visualized by ECL solution (Amersham, Sweden).

### 2.9. Statistical Analysis

Data were all expressed as the mean ± SD. Multiple comparisons were performed by two-way ANOVA. Comparisons for two groups were performed by using a Student's *t*-test (GraphPad Prism 5 software). *p* < 0.05 was considered to be significant.

## 3. Results

### 3.1. The EC50 Values of APAP and H_2_O_2_

As shown in Table 1, EC50 values of APAP or H_2_O_2_ on BRL-3A and HL-7702 hepatocytes were calculated by MTT assay. EC50 value of APAP and H_2_O_2_ on BRL-3A cells was 6.4 ± 1.3 mM and 480 ± 96 *μ*M, respectively, and on HL-7702 cells was 8 ± 1.6 mM and 600 ± 120 *μ*M. The EC50 values of APAP and H_2_O_2 _were used for producing in vitro hepatocyte injury models.

### 3.2. The Characteristics of HST-T6-MVs and LX-2-MVs

**Figure 1:**
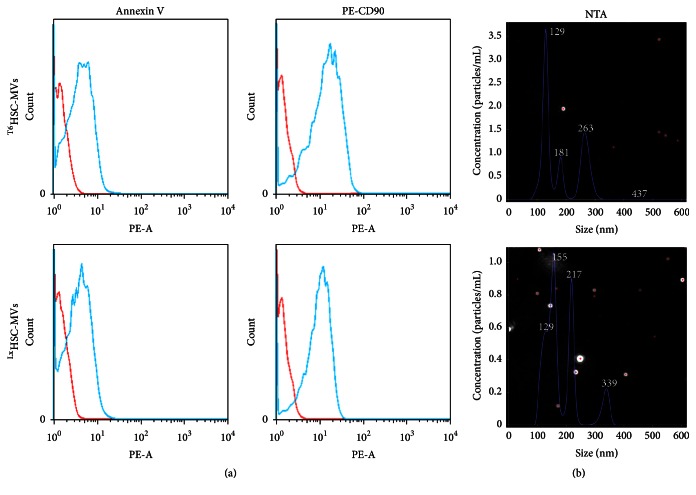
^T6^HSC-MVs and ^Lx^HSC-MVs separation and characterization. (a) Flow cytometric showed that HSC-MVs were specific stained with PE-CD90 and annexin V. (b) NTA analysis measured the size and amout of HSC-MVs.

Flow cytometric analysis indicated that both ^T6^HSC-MVs and ^Lx^HSC-MVs expressed annexin V and HSCs specific marker CD90 (Figure 1(a)). Nanoparticle tracking analysis (NTA) analysis showed that HSC-MVs were in size of 100 nm to 400 nm, and the concentration of HSC-MVs was about 2 × 10^10^/30 mL cell culture medium (Figure 1(b)).

### 3.3. HSC-MVs Increase the Cell Viability in BRL-3A and HL-7702 Hepatocyte Injury Models

**Figure 2:**
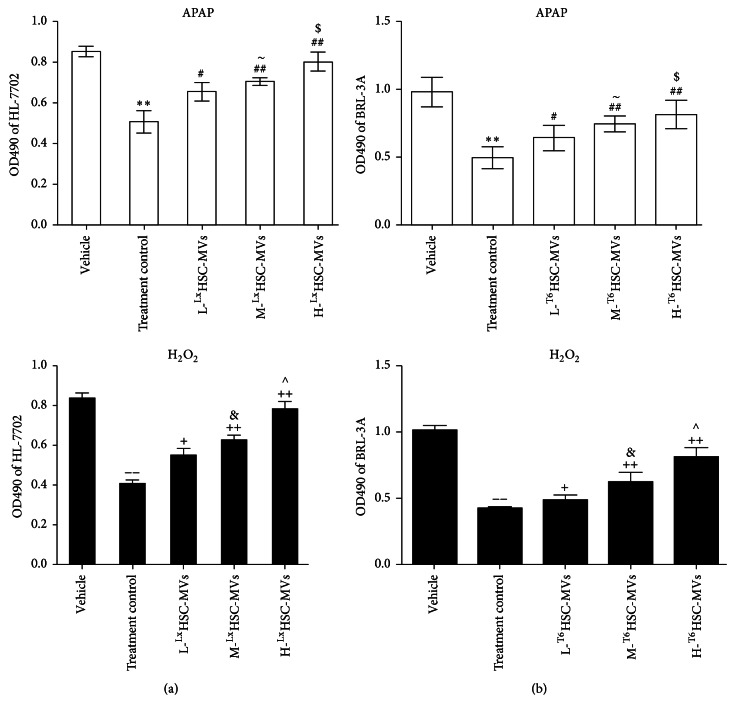
HSC-MVs increased the proliferation of HL-7702 and BRL-3A. (a) Proliferation of APAP or H_2_O_2_-treated BRL-3A cocultured with ^T6^HSC-MVs. (b) Proliferation of APAP or H_2_O_2_-treated HL-7702 cocultured with ^Lx^HSC-MVs. ^*∗∗*^ *p* < 0.01 versus vehicle, APAP; ^−−^ *p* < 0.01 refers to M-HSC-MVs versus vehicle, H_2_O_2_; ^#^ *p* < 0.05, ^##^ *p* < 0.01 versus treatment control, APAP; ^+^ *p* < 0.05, ^++^ *p* < 0.01 versus positive control, H_2_O_2_; ^~^ *p* < 0.01 refers to M-HSC-MVs versus L-HSC-MVs, APAP; ^&^ *p* < 0.05 refers to M-HSC-MVs versus L-HSC-MVs, H_2_O_2_; ^$^ *p* < 0.05 versus M-HSC-MVs, APAP; ^∧^ *p* < 0.05 versus M-HSC-MVs, H_2_O_2_; *n* = 3/group; ~ represents *p* < 0.05 versus L-HSC-MVs, APAP; & represents *p* < 0.05 versus L-HSC-MVs, H_2_O_2_.

MTT cell proliferation assay showed that both BRL-3A and HL-7702 hepatocytes were injured after APAP or H_2_O_2_ treatment for 48 h (treatment control versus vehicle; Figures 2(a) and 2(b)). We found that HSC-MVs significantly increased the proliferation ability of APAP/H_2_O_2_-treated hepatocytes (versus treatment control; Figures 2(a) and 2(b)). In addition, our results demonstrated a dose-response effect of HSC-MVs in promoting the proliferation of BRL-3A and HL-7702. The viability of M-HSC-MVs treated cells was higher than L-HSC-MVs treated cell group (versus L-HSC-MVs; Figures 2(a) and 2(b)), and H-HSC-MVs treated hepatocytes had the highest cell viability (versus M-HSC-MVs; Figures 2(a) and 2(b)).

### 3.4. HSC-MVs Reduce the Apoptosis of BRL-3A and HL-7702 Hepatocyte Injury Models

**Figure 3:**
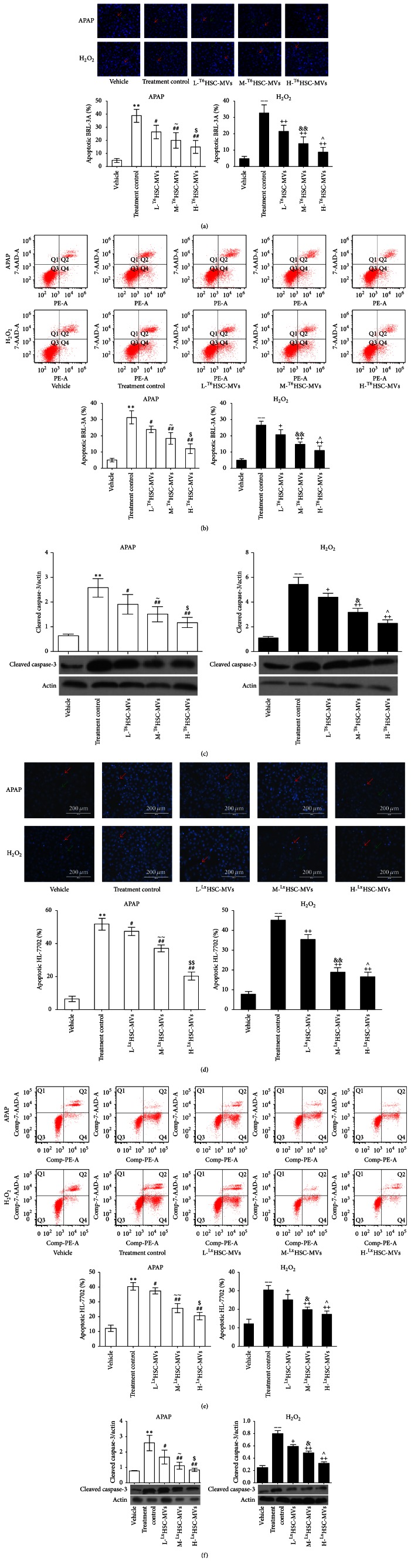
Effect of HSC-MVs on APAP or H_2_O_2_-treated BRL-3A and HL-7702 apoptosis and cleaved caspase-3 expression. (a) BRL-3A apoptosis monitored by Hoechst 33258 staining (red arrows represent apoptotic cells, green arrows represent normal cells). (b) BRL-3A apoptosis determined by flow cytometric analysis. (c) Expression of cleaved caspase-3 protein in BRL-3A. HL-7702 apoptosis detected by Hoechst 33258 staining (d) and flow cytometric analysis (e). (f) Expression of cleaved caspase-3 protein in HL-7702. ^*∗∗*^ *p* < 0.01 versus vehicle, APAP; ^−−^ *p* < 0.01 versus vehicle, H_2_O_2_; ^#^ *p* < 0.05, ^##^ *p* < 0.01 versus treatment control, APAP; ^+^ *p* < 0.05, ^++^ *p* < 0.01 versus treatment control, H_2_O_2_; ^~~^ *p* < 0.01 versus L-HSC-MVs, APAP; ^&&^ *p* < 0.01 versus L-HSC-MVs, H_2_O_2_; ^$^ *p* < 0.05, ^$$^ *p* < 0.01 versus M-HSC-MVs, APAP; ^∧^ *p* < 0.05 versus M-HSC-MVs, H_2_O_2_; *n* = 3/group; ~ represents *p* < 0.05 versus L-HSC-MVs, APAP; & represents *p* < 0.05 versus L-HSC-MVs, H_2_O_2_.

Hoechst 32258 staining and annexin V-PE/7-AAD analysis revealed that both APAP and H_2_O_2_ induced apoptosis in BRL-3A and HL-7702 cells (versus vehicle; Figures 3(a), 3(b), 3(c), and 3(d)). Treatment with HSC-MVs markedly reduced the cell apoptosis rate and cleaved caspase-3 protein level when compared with untreated group (versus treatment control; Figures 3(a), 3(b), 3(c), 3(d), 3(e), and 3(f)). Meanwhile, the protective effect of HSC-MVs was dose-dependent. The apoptosis rate and cleaved caspase-3 expression of M-HSC-MVs treated cells were lower compared with L-HSC-MVs treated cells (versus L-HSC-MVs; Figures 3(a), 3(b), 3(c), 3(d), 3(e), and 3(f)), while the H-HSC-MVs had the most protective effect on the injured hepatocytes (versus M-HSC-MVs; Figures 3(a), 3(b), 3(c), 3(d), 3(e), and 3(f)).

### 3.5. HSC-MVs Reduce the Levels of AST, ALT, and LDH in the Culture Media of BRL-3A and HL-7702 Hepatocyte Injury Models

**Figure 4:**
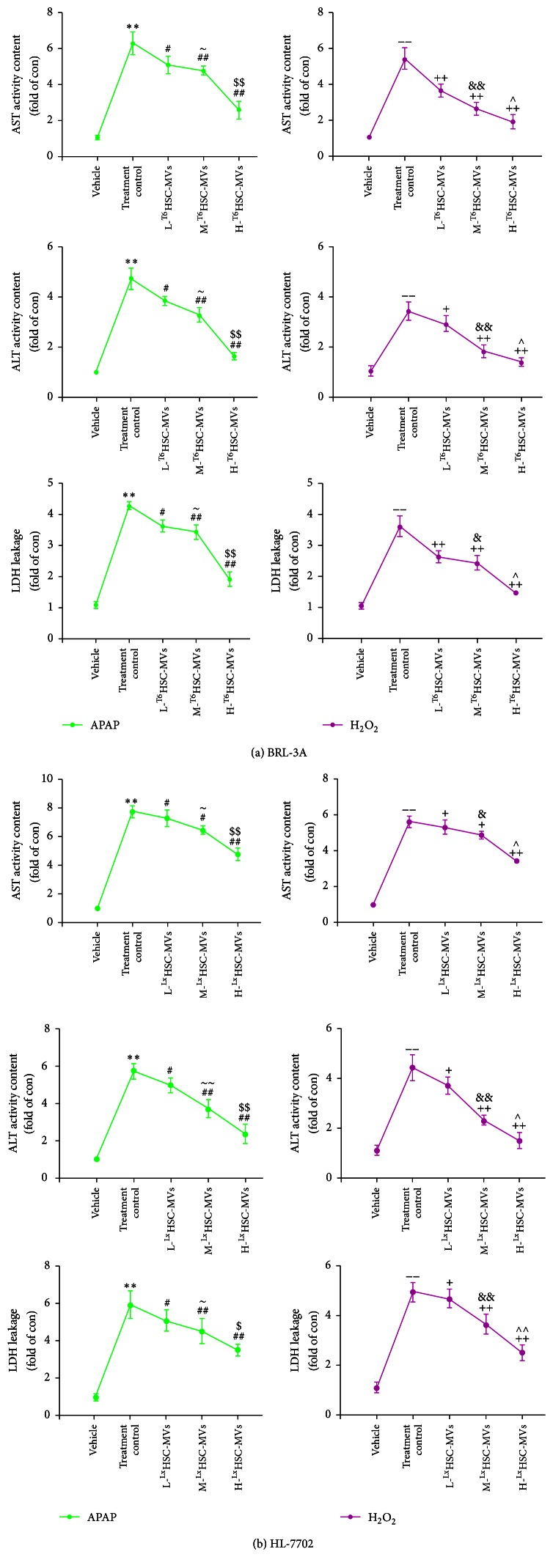
Effect of HSC-MVs on AST, ALT, and LDH leakage of APAP or H_2_O_2_-treated BRL-3A and HL-7702. (a) AST, ALT, and LDH leakage of APAP or H_2_O_2_-treated BRL-3A cocultured with ^T6^HSC-MVs. (b) AST, ALT, and LDH leakage of APAP or H_2_O_2_-treated HL-7702 cocultured with ^Lx^HSC-MVs. ^*∗∗*^ *p* < 0.01 versus vehicle, APAP; ^−−^ *p* < 0.01 versus vehicle, H_2_O_2_; ^#^ *p* < 0.05, ^##^ *p* < 0.01 versus treatment control, APAP; ^+^ *p* < 0.05, ^++^ *p* < 0.01 versus treatment control, H_2_O_2_; ^~~^ *p* < 0.01 versus L-HSC-MVs, APAP; ^&^ *p* < 0.05, ^&&^ *p* < 0.01 versus L-HSC-MVs, H_2_O_2_; ^$^ *p* < 0.05, ^$$^ *p* < 0.01 versus M-HSC-MVs, APAP; ^∧^ *p* < 0.05, ^∧∧^ *p* < 0.01vs M-HSC-MVs, H_2_O_2_; *n* = 3/group; ~ represents *p* < 0.05 versus L-HSC-MVs, APAP.

Our result showed that the levels of AST, ALT, and LDH in the culture media of BRL-3A and HL-7702 hepatocyte were significantly increased after being treated with APAP or H_2_O_2_ for 24 h (versus vehicle; Figure 4). In HSC-MVs treated BRL-3A and HL-7702 cells, the leakage of AST, ALT, and LDH was significantly decreased (versus treatment control; Figures 4(a) and 4(b)). We also found that protective effect of HSC-MVs was dose-dependent (H-HSC-MVs versus M-HSC-MVs; M-HSC-MVs versus L-HSC-MVs; Figures 3(a), 3(b), 3(c), and 3(d)).

## 4. Discussion

This study evaluated the role of MVs derived from HSCs in attenuating xenobiotic-induced liver injury. For this purpose, we explored the hepatoprotective effect of HSC-MVs in APAP and H_2_O_2_ induced hepatocyte (BRL-3A or HL-7702) injury models. HSCs are liver-specific mesenchymal cells, homing between the sinusoidal endothelial cells and hepatic epithelial cells [27]. HSCs express classical mesenchymal makers (CD105, CD44, CD29, CD13, and CD90), but not for the endothelial marker CD31, endothelial progenitor cell marker CD133, or hematopoietic markers (CD45 and CD34). LX-2 and HST-T6 are HSCs [22]. The MVs derived from HSC-T6 and LX-2 cells were confirmed by detecting the expression of CD90 (HSC specific marker) and annexin V (MVs specific marker) [28]. More important, we found that HSC-MVs dose-dependently increased hepatocyte viability and decreased cell apoptosis in both liver injury models. The protective effect was also evidenced by the ability of HSC-MVs in inhibiting ALT, AST, and LDH leakage of hepatocytes induced by APAP and H_2_O_2_.

MVs are submicron membrane fragments released from virtually all cell types and participated in regulating various functions of the target cells [29]. Stem cells-derived MVs have been reported to participate in the repair of various tissue injuries [30, 31]. In the present study, we first reported the therapeutic effects of HSC-MVs on xenobiotics injured hepatocytes. APAP and H_2_O_2_ are two well-known hepatotoxicants which mediate liver injury. APAP and H_2_O_2 _represent two different mechanisms of liver toxicity. APAP causes liver injury by both covalent modification of protein targets and oxidative stress mediated injury pathway, whereas H_2_O_2_ mediates liver injury individually through the oxidative stress pathway [6, 32]. Both APAP and H_2_O_2_ induce apoptosis of hepatocytes [33, 34]. Therefore, APAP and H_2_O_2_ were used to build in vitro liver injury models in this study. Our results showed that MVs derived from LX-2 and HST-T6 dose-dependently promoted cell viability and inhibited cell apoptosis and cleaved caspase-3 expression in our model systems. These findings are supported by previous reports showing HSCs to repair the injured liver [10, 14]. Our findings revealed that HSC-MVs can exert the therapeutic effect of their parent cells, which add novel therapeutic mechanisms of HSCs. Of note, our data indicate that HSC-MVs could have some privileges as a novel therapeutic avenue for treating hepatocyte injury because they less likely trigger immune response and in vivo tumorigenesis.

Liver enzymes, especially ALT, AST, and LDH, are routinely measured in screening assays for the detection of liver injury [25, 26, 35]. The leakages of LDH, AST, and ALT are significantly increased in hepatocytes exposed to hepatotoxicant [36]. In this study, we found that ALT, AST, and LDH released from HL-7702 and BRL-3A hepatocytes were significantly increased after the hepatocytes were exposed to APAP or H_2_O_2_. Consistent with our expectation, MVs had a dose-dependent effect in decreasing the medium level of ALT, AST, and LDH. This is consistent with a recent in vivo study in which exosomes derived from mesenchymal stem cell have been found to decrease the biochemical parameters (ALT and AST) and hepatocyte injury induced by CCl4 [6]. These results provide further evidence to support the protective effect of HSC-MVs on hepatocyte injury. Whether this effect is linked to MV membrane merge/fusion with the membrane of injured cells remains to be further investigated.

## 5. Conclusion

In conclusion, treatment with HSC-derived MVs can protect hepatocytes from toxicant-induced injury. The beneficial effects of MVs are likely through maintaining the proliferative activity and the antiapoptotic and antiautophagy abilities of hepatocytes. HSC-MVs may present a new therapy in drug-induced liver toxicity, in lieu of the limited availability of the conventional liver transplants. However, further investigations are needed to determine the hepatoprotective components of the MVs and the exact molecular mechanisms of the action.

## Figures and Tables

**Table 1:** The EC50 values of APAP and H_2_O_2_ in inhibiting BRL-3A and HL-7702 proliferation determined by MTT analysis.

Cell types	Chemicals	
	APAP (mM)	H_2_O_2_ (*μ*M)
BRL-3A, MTT EC50	6.4 ± 1.6	480 ± 120
HL-7702, MTT EC50	8 ± 1.6	600 ± 120

EC50: concentration of APAP/H_2_O_2_ that induces MTT reduction by 50%. Values were calculated by using linear statistical regression analysis. *N* = 6/group.
